# Nutritional Profiling and Preliminary Bioactivity Screening of Five Micro-Algae Strains Cultivated in Northwest Europe

**DOI:** 10.3390/foods10071516

**Published:** 2021-07-01

**Authors:** Joran Verspreet, Lise Soetemans, Caoimhe Gargan, Maria Hayes, Leen Bastiaens

**Affiliations:** 1 Flemish Institute for Technological Research (VITO), 2400 Mol, Belgium; joran.verspreet@vito.be (J.V.); lise.soetemans@vito.be (L.S.); 2 Teagasc Ashtown Food Research Centre, Food Biosciences Department, D15 KN3K Dublin, Ireland or caoimhegargan@icloud.com (C.G.); Maria.Hayes@teagasc.ie (M.H.)

**Keywords:** digestibility, bioactivity, micro-algae, nutritional profile, cell disruption

## Abstract

This study aimed to map the nutritional profile and bioactivities of five microalgae that can be grown in Northwest Europe or areas with similar cultivation conditions. Next to the biochemical composition, the in vitro digestibility of carbohydrates, proteins, and lipids was studied for *Chlamydomonas nivalis*, *Porphyridium purpureum*, *Chlorella vulgaris*, *Nannochloropsis gaditana*, and *Scenedesmus* species biomass. These microalgae were also assessed for their ability to inhibit the angiotensin-1-converting enzyme (ACE-1, EC 3.4.15.1), which is known to play a role in the control of blood pressure in mammals. Large differences in organic matter solubility after digestion suggested that a cell disruption step is needed to unlock the majority of the nutrients from *N. gaditana* and *Scenedesmus* species biomass. Significant amounts of free glucose (16.4–25.5 g glucose/100 g dry algae) were detected after the digestion of *C. nivalis*, *P. purpureum*, and disrupted *Scenedesmus*. The fatty acid profiles showed major variations, with particularly high Ω-3 fatty acid levels found in *N. gaditana* (5.5 ± 0.5 g/100 g dry algae), while lipid digestibility ranged from 33.3 ± 6.5% (disrupted *N. gaditana*) to 67.1 ± 11.2% (*P. purpureum*). *C. vulgaris* and disrupted *N. gaditana* had the highest protein content (45–46% of dry matter), a nitrogen solubility after digestion of 65–71%, and the degree of protein hydrolysis was determined as 31% and 26%, respectively. Microalgae inhibited ACE-1 by 73.4–87.1% at physiologically relevant concentrations compared to a commercial control. These data can assist algae growers and processors in selecting the most suitable algae species for food or feed applications.

## 1. Introduction

Micro-algae, further referred to as algae, have emerged as a sustainable and nutritious feedstock for food and feed [1,2]. The CO_2_-capturing capacity of autotrophic algae and the possibility of cultivating them without using fertile soil and with limited use of water, enables sustainable algae cultivation. They are an attractive source of nutrients such as proteins and long-chain polyunsaturated fatty acids (PUFAs), including eicosapentaenoic acid (EPA, C20:5) and docosahexaenoic acid (DHA, C22:6) [3]. Specific algae species are, together with fish, the sole sources in the human diet of EPA and DHA, which both serve important physiological functions in the body [4]. Next to nutrient levels, nutrient digestibility and accessibility are also relevant in this context. Although research has been carried out on the digestibility of algae proteins [5,6,7,8] and lipids [9,10], carbohydrate digestibility has received little attention. Carbohydrates are, nevertheless, a major algae constituent [11] and have the potential to provide a significant amount of energy. In some studies [12], the total pool of soluble carbohydrates after digestion was measured. However, this only provides a very rough estimation of carbohydrate digestibility. Indeed, it can be hypothesized that a significant portion of soluble carbohydrates is indigestible. Algae carbohydrates are very diverse in terms of composition and linkage type [11], making them less likely to be hydrolyzed by the limited set of carbohydrate-degrading enzymes in the upper part of the gastrointestinal tract. A more accurate evaluation of carbohydrate digestibility is hence preferable. Good knowledge of the biomass’ post-cultivation treatment is also important in this regard, as processing can lead to cell disruption, and hence affect nutrient release and digestibility.

Most studies on the nutritional properties of algae have only focused on the protein or lipid fraction, but there is a need for more complete characterization. Such an analysis is required, amongst others, to facilitate the incorporation of algae in animal feed. Indeed, feed formulations are precisely balanced on many (more) different levels, with specific targets for essential nutrients and energy content. An exhaustive analysis, including the characterization of minor constituents, is also needed for the optimization of a fractionation process where minor constituents and remaining fractions must also be valorized to the maximum.

The aim of this research was, therefore, to study nutrient composition together with the digestibility of proteins, lipids, and carbohydrates and to perform a preliminary bioactivity screening. For the latter, the angiotensin-1-converting enzyme (ACE-1; EC3.4.15.1) inhibition activity was tested because of earlier promising results with *Spirulina platensis* and *Isochrysis galbana* biomass [13]. ACE-1 is a key enzyme that helps to regulate the salt–water balance and blood pressure within the renin angiotensin aldosterone system found in humans and some animals, including dogs. Furthermore, the level of free orthophosphate after digestion was determined to obtain the first indication of phosphorous bio-accessibility. This was carried out for a set of algae that can be grown in the same climate zone, i.e., Northwest Europe, or more in general, in an oceanic climate zone with a similar light incidence as in Northwest Europe. Such a comparison is particularly interesting for the algae grower who is bound to the prevailing climate conditions. To evaluate digestibility, the human digestion was simulated in vitro.

## 2. Materials and Methods

### 2.1. Algae Biomass

*Chlorella vulgaris* and *Scenedesmus* biomass were supplied by the institute of Bio- and Geosciences (IBG-2), Forschungszentrum Jülich (Jülich, Germany). Microscopy analysis indicated that the *C. vulgaris* culture contained a limited level (< 20%) of *Scenedesmus* sp. cells. This culture was harvested by membrane filtration, which included a desalting step.

The *C. vulgaris* salty culture (2.5% NaCl) was grown in closed bioreactors and was harvested and desalted at VITO by low shear membrane filtration.

The *Scenedesmus* culture was most likely *Scenedesmus obliquus*, but is referred to as *Scenedesmus* throughout the manuscript. It was grown in a fresh medium (no NaCl added) in closed bioreactors and harvested by centrifugation. *Chlamydomonas nivalis*, *Porphyridium purpureum*, and *Nannochloropsis gaditana* were cultivated and harvested at the Sunbuilt facility (Thomas More/Vito, Geel, Belgium) in 300–1500 L closed photobioreactors. *C. nivalis* was cultivated in a fresh medium, while *P. purpureum* and *N. gaditana* were cultivated in a brackish medium (1.2% *w/v* NaCl).

All algae concentrates were freeze dried and stored at −20 °C. As explained in Section 3, *N. gaditana* and *Scendesmus* samples were also disrupted by bead milling (DYNO^®^-MILL-Mulitlab, Spring Lake, MI, USA). Milling was performed in a 0.514 L milling chamber filled to 64.2% (volume-based) with 0.5 mm beads (YTZ^®^ grinding media, Tosoh, Tokyo, Japan). The pump and mill settings for *Scenedesmus* and *N. gaditana* were set so that the average residence time was 24 and 6 min, respectively. The settings were optimized in previous experiments with the aim of maximum organic matter solubility after milling with a minimum residence time.

### 2.2. Biochemical Analysis

Dry matter content was evaluated by weighing samples before and after overnight drying at 105 °C, while ash content was determined after subsequent drying at 550 °C (4 h). The total lipid content was measured by chloroform:methanol extraction according to the procedure of Ryckebosch et al. [14]. For the analysis of the fatty acid composition, the fatty acids in the lipid extract were methylated under sequential alkaline and acid conditions following the ISO 12966-2:2011 protocol [15]. Fatty acid methyl esters (FAMEs) were quantified by gas chromatography–flame ionization detection (GC-FID) using a TR-FAME column (100 m length, 0.25 mm internal diameter, 0.20 µm film thickness, Thermo Scientific, Waltham, MA, United States). Amino acid analysis was performed by high-performance anion-exchange chromatography with pulsed amperometric detection (HPAEC-PAD) after acid hydrolysis with phenol-HCl (6 N, 23 h, 110 °C) according to ISO protocol 13903:2005. Amino acid separation was performed using a Dionex AminoPac PA-10 column (2 nm × 250 nm) and a previously detailed elution gradient [16]. Tryptophane was not analyzed due to its liability for acid degradation. Only the sum of glutamate and glutamine was measured, because the latter is converted to the first during acid hydrolysis. Similarly, the sum of aspartate and asparagine was measured, and not their individual levels. The total protein content was calculated as the sum of all amino acids each corrected for water uptake during hydrolysis. The carbohydrate content was measured after a two-step H_2_SO_4_ hydrolysis and HPAEC-PAD analysis, as described before [17]. The total carbohydrate level was estimated by summing monosaccharides and uronic acids, each corrected for water uptake during hydrolysis. For mineral analysis, algae (0.4 g) were mixed with AgNO_3_ (1 M, 1 mL) and HNO_3_ (5 mL, 67–69%), incubated for 2 h at 105 °C and transferred to a new tube. The sample was diluted with ultrapure water to a volume of 15 mL, centrifuged (20 min, 4000 rpm) and the supernatant was transferred to another tube. The washing step was executed two more times by adding 15 mL of water, shaking, centrifugation, and collecting the supernatant. The volume of the combined supernatant phases was adjusted to 50 mL with water, after which, the mixture was analyzed by inductively coupled plasma atomic emission spectroscopy (Thermo iCAP analyzer, Thermo Scientific) for P, K, Ca, Na, and Fe content. The precipitate was incubated with 1 mL of NH_3_ (25% m/m) in the dark and shaken. This was repeated until the precipitate turned into a solution. Next, the dissolved material was transferred to the first tube that was used for the AgNO_3_/HNO_3_ incubation. After dilution with water, the diluted product was analyzed for Cl content with high resolution inductively coupled plasma mass spectrometry (Thermo Element 2, Thermo Scientific). The latter was performed after calibration using 0-250-500-1000 µg Cl^−^/L solutions in 0.1% NH_3_ (*v/v*). Following the same protocol as above, a quality control was prepared with 1000 mg Cl^−^/L to verify that recovery was at least 95%. The Cl analysis was based on the procedure of Naozuka et al. [18].

### 2.3. In Vitro Digestibility

The standardized in vitro digestion protocol proposed by Minekus et al. [19] was used to evaluate nutrient digestibility. Blanks containing digestive fluids, enzymes, and bile but no algae, were included in each experiment to account for nutrients not coming from algae digestion. Digestion tests were preceded by pH adjustment experiments to accurately determine the amount of NaOH and HCl needed to reach the pH values prescribed in the consensus protocol [19]. As recommended by Brodkorb et al. [20], the substrate:fluid ratio for oral phase simulation was adapted in order to obtain a swallowable bolus with a paste-like consistency. In fact, substrate:fluid ratios of 1:5 and 1:2 were used for *P. purpureum* and the other algae samples, respectively. Water was added to reach the standard mass at the start of digestion.

For the evaluation of protein digestibility, the digestion was stopped by adding a protease inhibitor (1 mM 4-(2-aminoethyl) benzenesulfonyl fluoride hydrochloride). Subsequently, the digesta was centrifuged (5 min, 5000× *g*) and supernatant aliquots were examined. Firstly, the total amount of nitrogen in the supernatant was measured with an automated Dumas protein analysis system (Vario EL Cube, Elementar, Langenselbold, Germany) and compared with the amount of nitrogen in the initial algae biomass. Secondly, the degree of protein hydrolysis was estimated by the quantification of the primary amino groups and comparison with the total nitrogen in the sample. Samples were incubated overnight in 2% sodium dodecyl sulfate solution at 4 °C, and added to a freshly prepared mixture of o-phthalaldehyde (OPA) and N-acetyl-cysteine (NAC) in borate buffer, as described before [21]. By using an L-isoleucine calibration series and absorbance measurement at 340 nm (Tecan Infinite 200 PRO, Tecan, Männedorf, Switzerland), the concentration of primary amino groups can be determined [22]. The degree of protein hydrolysis was calculated as the ratio of primary amino groups to the total algae nitrogen. A correction was made for primary amino groups and total nitrogen in the blank.

For the assessment of the lipid digestibility, digestion was ended by adding chloroform:methanol, as proposed by Brodkorb et al. [20], immediately followed by lipid extraction, as described above. In case lipid extraction could not be executed instantly after digestion, a lipase inhibitor was added (4-bromophenylboronic acid in methanol, 5 mM inhibitor/mL digesta), the digesta was snap frozen, stored at −20 °C and extracted with chloroform:methanol on another day. After lipid extraction, the amount of free fatty acids (FFAs) was determined after their derivatization to fatty acid diethylamides and subsequent GC-FID analysis [23]. A famewax column (30 m length, 0.32 mm internal diameter, 0.25 µm film thickness, Restek, Bellefonte, PA, USA) was used for GC separation, pentadecanoic acid (C15:0) as the internal standard, and a lauric acid (C12:0) dilution series for detector calibration.

Carbohydrate digestibility was tested by executing the standard digestion protocol followed by centrifugation (5 min, 5000× *g*) and incubation with *Aspergillus niger* amyloglucosidase (A1602 Sigma Aldrich, Munich, Germany), which is needed to simulate the activity of intestinal brush border enzymes [19]. To this end, 1 mL of supernatant was transferred to a new recipient, the pH adapted to 4.5 and *A. niger* amyloglucosidase (12 U) was added. Next, the mixture was incubated for 60 min at 37 °C, heated (10 min at 95 °C) to inactivate enzymes, and the hydrolysate diluted and analyzed by HPAEC-PAD, as described above.

Finally, the concentration of dissolved orthophosphate in the digesta was measured. After the heat inactivation of enzymes (10 min at 95 °C), the digesta was cooled to room temperature and diluted so that dissolved orthophosphate could be measured with either the LCK348 or LCK 350 total/ortho phosphate kit (Hach Lange, Düsseldorf, Germany) according to the manufacturer’s instructions.

### 2.4. Angiotensin-1-Converting Enzyme (ACE-1; EC 3.4.15.1) Inhibition Assay 

Assessment for the ability of the microalgae to inhibit the ACE-1 enzyme was carried out using the ACE-I inhibition bioassay using a previously published method [13,24]. The ACE-1 inhibition kit (ACE-1 inhibition kit—WST, Dojindo Laboratories, Kumamoto, Japan) was used. In brief, 20 µL of each sample prepared at a concentration of 1 mg/mL in water was added to 20 µL of the substrate and 20 µL of the enzyme working solution in triplicate. Captopril^©^ was used as a positive control at a concentration of 0.05 mg/mL dissolved in ddH_2_O. Samples were incubated at 37 °C for 1 h. An amount of 200 μL of indicator working solution was then added to each well, and subsequent incubation at room temperature was carried out for 10 min. Absorbance at 450 nm was read using a FLUOstarOmega microplate reader (BMG LABTECH GmbH, Offenburg, Germany). The percentage ACE-1 inhibition was calculated using the following equation:

ACE-I inhibition (%) = ((A_0_ − A_I_)/A_0_) × 100% (1)

where *A*_0_ is the substrate absorbance at 450 nm in the presence of ACE-I and absence of the inhibitor, and *A*_I_ is the substrate absorbance at 450 nm in the presence of ACE-I and the inhibitor or Captopril^©^ (positive control).

### 2.5. Statistics

Statistical analyses were performed with Statistica version 12 (Dell Inc., Tulsa, OK, USA, 2015) with 5% as the significance threshold level. One-way ANOVA was used to evaluate the impact of algae type on digestibility parameters. After a positive omnibus test, a post hoc Tukey-test was performed.

## 3. Results and Discussion

### 3.1. Sample Pretreatment and Biochemical Composition

When algae are cultivated for food or feed applications, it is important that nutrients do not remain physically locked in the cell during digestion. In that case, a cell disruption step may be appropriate so that the algae’s nutritional potential is exploited to the maximum.

A preliminary test was therefore performed to identify for which algae the cell structure could have such a nutrient-encapsulating effect. Algae were digested in vitro and the amount of soluble organic matter was determined. Since carbohydrates, proteins, and lipids can only be absorbed in the small intestine when they are degraded to smaller building blocks that are soluble in the liquid/micellar phase, a low organic matter solubility after digestion can be seen as an indication for a low combined digestibility of carbohydrates, proteins, and lipids.

**Figure 1:**
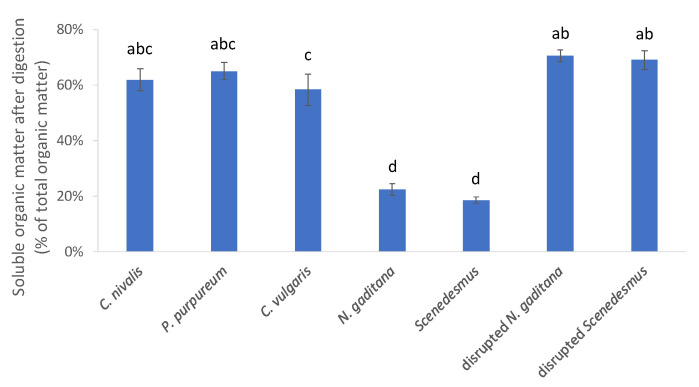
Organic matter soluble after digestion, expressed as a percentage of the initial organic matter level. Values that are not labeled with a common letter are significantly different.

For *C. nivalis*, *P. purpureum* and *C. vulgaris*, around 60% of the organic matter was soluble after in vitro digestion, while for *N. gaditana* and *Scenedesmus*, only about 20% of the organic matter was soluble after digestion (Figure 1). This suggests that the majority of the organic fraction of *N. gaditana* and Scenedesmus biomass cannot be absorbed in the small intestine.

This accords with earlier observations showing that none or only a negligible fraction of the lipids and proteins are bio-accessible in non-disrupted *Nannochloropsis oculata* [10] and that cell disruption is crucial to make lipids [9,10] and carotenoids [9] bio-accessible from *Nannochloropsis* cells. *Scenedesmus* sp. have a notoriously recalcitrant cell wall, and heating to 90 °C [25], ultrasound treatment [26], or a combination of a mechanical and enzymatic treatment [27] were previously needed to significantly disrupt Scenedesmus cells and to solubilize the cell content. When the Scenedesmus and *N. gaditana* biomass were disrupted by bead milling before digestion in the current study, the soluble organic matter fraction increased to 68.9 ± 3.5% and 70.5 ± 2.2%, respectively (Figure 1). As these data indicate that a disruption step is needed to make considerable levels of nutrients susceptible to digestion and available for absorption, it was decided to only characterize the disrupted *N. gaditana* and *Scenedesmus* samples in the further course of the study.

Due to the high sample amounts needed, it was decided to use a new batch of disrupted *N. gaditana* from this point onwards. This new batch had a soluble organic matter after digestion of 62.8 ± 0.8%.

As shown in Table 1, carbohydrates were important algae constituents for *C. nivalis*, *P. purpureum*, and disrupted *Scenedesmus* and the sum of all monosaccharides and uronic acids ranged between 38% and 51% of the dry matter (dm). Proteins were the main constituent of disrupted *N. gaditana* and *C. vulgaris* biomass. Lipid levels ranged between 13.2% (*P. purpureum*) and 31.9% (disrupted *N. gaditana*). The digestibility of these macronutrients is discussed below.

### 3.2. Carbohydrate Composition and Digestibility

**Figure 2:**
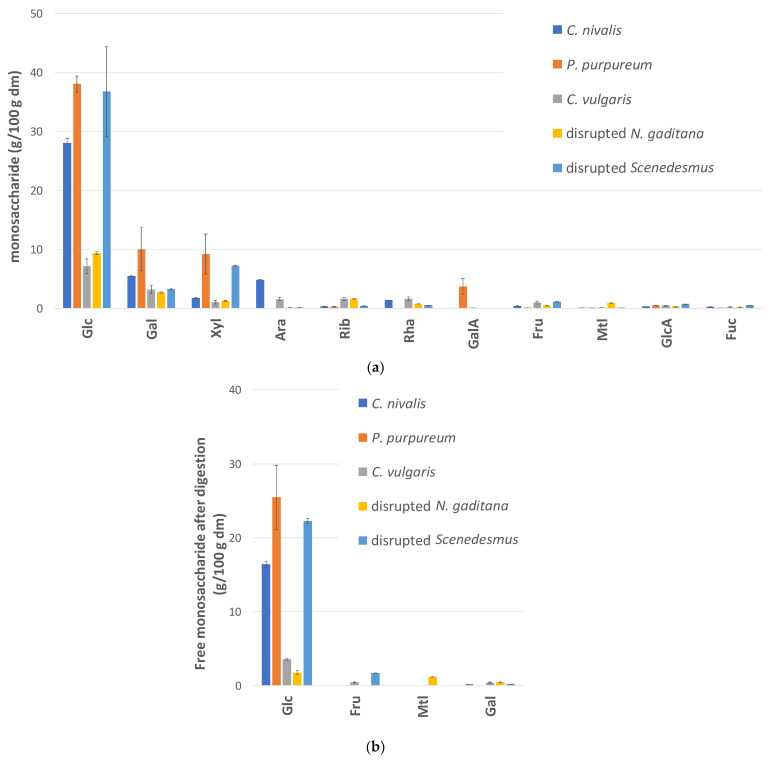
(**a**) Total monosaccharide and uronic acid levels in the initial biomass measured by anion exchange chromatography after acid hydrolysis. (**b**) Free monosaccharides in the liquid digesta after digestion measured by HPAEC. Glc = glucose, Gal = galactose, Xyl = xylose, Ara = arabinose, Rib = ribose, Rha = rhamnose, GalA = galacturonic acid, Fru = fructose, Mtl = mannitol, GlcA = glucuronic acid, Fuc = fucose. Bars and error bars represent averages and standard deviations, respectively. These data are also included in Supplementary Materials Table S1.

The monosaccharide composition analysis (Figure 2a) shows that glucose was the main monosaccharide in all algae samples, with concentrations ranging between 7.1% and 38.1% on a dm basis. Other monosaccharides, mainly galactose, xylose, and arabinose, were present in lower concentrations (Figure 2a).

Carbohydrates serve various functions in the algae cell, including building up the cell structure and serving as an energy stock. Glucose can be part of the structural carbohydrates, such as the extracellular polysaccharides in *Porphyridium* sp. [28,29] or as cellulose as in *N. gaditana* [29,30]. Additionally, glucose is also part of glucose-based storage polysaccharides. *C. nivalis* is known to accumulate starch [31,32], and starch levels up to 35% and 39% on a dm basis were reported for *Chlamydomonas reinhardtii* [33,34] and heterotrophically cultivated *Scenedesmus* sp. [35,36], respectively. Only 2.1% of floridean starch was found in a commercial *P. purpureum* sample [29] but levels might be higher under different growth conditions, as seen for *C. vulgaris* [37,38]. Indeed, low levels (0–14%) of α-linked glucose were found in commercial *Chlorella* sp. [6] and *C. vulgaris* [29] products but starch levels of 30–35% [39] and 50% [40] were obtained for *C. vulgaris* cultivated under conditions favoring starch accumulation. *N. gaditana* differs from the other algae species in that it contains no or negligible amounts of starch [41], while it synthesizes chrysolaminarin, a storage polymer composed of β-linked glucose units [42].

When the algae fractions underwent in vitro digestion, significant amounts of free glucose (16.4–25.5 g glucose/100 g dry algae) were detected in the case of *C. nivalis*, *P. purpureum*, and disrupted *Scenedesmus* (Figure 2b). The digestion of *C. vulgaris* and disrupted *N. gaditana* yielded only 3.6 g free glucose/100 g dm and 1.8 g free glucose/100 g dm, respectively. This was expected, since these algae had a much lower initial glucose content.

These data do not allow a precise prediction of the in vivo glucose release, since a detailed kinetic study of starch degradation was not performed and because of the limitations of in vitro digestion studies [20]. Yet, these results do suggest that considerable glucose levels can become available for intestinal uptake during the digestion of *C. nivalis*, *P. purpureum* and disrupted *Scenedesmus*, and that glucose will contribute significantly to the energy content. It is noteworthy that algae biomass was not heated before digestion. One would expect that algal starch was not yet gelatinized and hence, partly resistant to hydrolysis by intestinal enzymes, as seen for other starch types such as cereal starches [43]. Indeed, in vitro digestion was performed at 37 °C, so below the gelatinization temperature of common food starches (50–70 °C) [44]. Yet, it is plausible that gelatinization occurs at lower temperatures for *P. purpureum* since the gelatinization of floridean starch in red macroalgae was reported to start already at 41–42 °C [45]. For disrupted *Scenedesmus*, the disruption step could have affected the degree of starch (semi-)crystallinity, which may increase its susceptibility to enzymatic degradation, as is the case, for example, for ball-milled microcrystalline cellulose [46]. Moreover, it cannot be excluded that endogenous algae enzymes also played a role in degrading (non-gelatinized) starch. For disrupted *N. gaditana*, the limited glucose release is not surprising, since the glucose units in chrysolaminarin are connected by β-linkages, which are not susceptible to degradation by human α-amylases or disaccharidases [47].

The concentrations of the other free monosaccharides (<2%) were much lower than that of glucose (Figure 2b). Galactose, fructose, and mannitol will have a negligible impact on energy supply. However, the latter two can still be relevant, as they belong to the so-called FODMAPs. FODMAPs are fermentable oligo-, di- and monosaccharides and polyols that are not or are incompletely absorbed in the small intestine. These compounds can cause gastro-intestinal problems with sensitive subjects upon consumption. Clinical trials indicated that eliminating FODMAPS from the diet relieves the symptoms of 50–80% of patients with irritable bowel syndrome with improvements in bloating, flatulence, diarrhea and general symptoms [48,49]. Yet, the fructose and mannitol amounts released during digestion of the studied algae (Figure 2b) are low compared with the levels previously reported to be mal-absorbed [49] and are probably too low to induce adverse effects.

### 3.3. Lipid Composition and Digestibility

To assess the nutritional value of the lipid fraction, the fatty acid composition was first evaluated (Table 2). The discussion focuses on the essential fatty acids linoleic acid (LA, (cis,cis-9,12)) and α-linolenic acid (ALA, C18:3 (cis 9,12,15)) on the one hand, and on EPA (C20:5) and DHA (C22:6) on the other hand. Since EPA and DHA synthesis by the human body is limited, several food authorities have set recommendations on their intake as well [4].

Linoleic acid was present in all algae fractions, with the highest levels in *C. vulgaris* (15.2 ± 0.8 mg/g dm), *C. nivalis* (12.6 ± 0.8 mg/g dm), and disrupted *Scenedesmus* (8.5 ± 0.5 mg/g dm). Additionally, α-linolenic acid was detected in these three algae, with levels ranging between 12.1 ± 0.6 mg/g dm (disrupted *Scenedesmus*), 22.4 ± 1.4 mg/g dm (*C. vulgaris*), and 27.0 ± 2.1 mg/g dm (*C. nivalis*). 

EPA was found in small amounts in *P. purpureum* (3.6 ± 0.8 mg/g dm) and was abundant in disrupted *N. gaditana* (54.9 ± 4.7 mg/g dm). DHA was only present in disrupted *N. gaditana* in low levels (0.20 ± 0.03 mg/g dm). The observed EPA concentrations are in line with earlier reported values for *N. gaditana* (3.4–4.3% dm) [50,51], *Nannochloropsis* sp. (1.1–6% dm) [52] and *P. purpureum* (0.07–1.6% dm) [53,54]. C16 and C18 fatty acids, especially C16:0, C18:1, C18:2, and C18:3, are known to dominate the fatty acid profiles of *C. vulgaris* [55,56], *C. nivalis* [57] and *Scendesmus* sp. [58,59].

Lipid digestibility was evaluated by comparing the concentration of free fatty acids (FFAs) after digestion with the total fatty acid content of the sample. It should be noted that this is not the same as the lipid bio-accessibility, i.e., the fraction available for uptake in the small intestine. Indeed, not only FFAs but also other lipid degradation products such as monoacylglycerols participate in the formation of micelles that are taken up by enterocytes in the gut. On the other hand, the incorporation of entirely hydrolyzed lipids in mixed micelles may be limited due to the presence of other cell compounds or due to the lipid localization in the cell [9].

**Figure 3:**
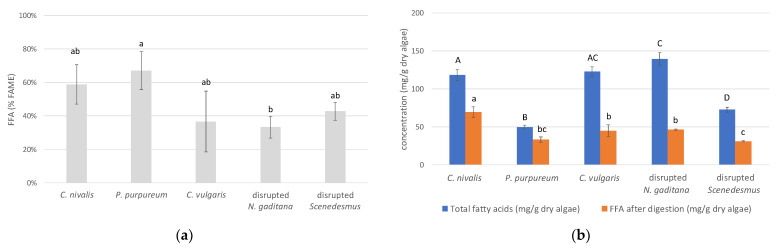
(**a**) Free fatty acids (FFAs) released from algae biomass after digestion, expressed as % of the total fatty acid content measured as fatty acid methyl esters (%FAMEs), and (**b**) expressed on dry algae basis. FFA levels within one graph are significantly different when they are not labeled with a common lowercase letter. Total fatty acid levels in the right graph are significantly different when they are not labeled with a common uppercase letter. A Tukey multiple comparison test was always used to compare values for different algae types.

*P. purpureum* and *C. nivalis* displayed the highest lipid digestibility, with 67.1 ± 11.2% and 58.9 ± 11.7% of the fatty acids liberated as free fatty acids after digestion, respectively (Figure 3a). Lower average values were observed for *C. vulgaris*, disrupted *N. gaditana* and disrupted *Scenedesmus* (36.6 ± 18.2%, 33.3 ± 6.5% and 42.8 ± 5.3%). *C. nivalis* had the highest absolute FFA content after digestion (69.6 ± 7.0 mg FFA/g dry algae, Figure 3b).

Due to the stereoselectivity of pancreatic lipases, only a limited fraction of the fatty acids can be liberated. Bernaerts et al. [9] estimated that in the case of *Nannochloropsis*, only 55–65% of the fatty acids can be released where this maximal hydrolysis value depends on the lipid classes (triacylglycerols, phospholipids, glycolipids) present in the biomass.

To our knowledge, no literature is available on the lipid digestibility of *C. nivalis* and *P. purpureum*. Sonication experiments with *Chlamydomonas reinhardtii* suggested that carotenoids are already fairly bio-accessible in *C. reinhardtii* without pre-treatment and that sonication has no additional effect [60]. Accordingly, a high lipid digestibility was found in this study for *C. nivalis* (58.9 ± 11.7%).

Regarding *C. vulgaris*, several studies, listed in a recently published review [11], showed that carotenoid bio-accessibility can be increased by pretreating the biomass by cell disruption. Yet, an enzymatic disruption could not increase chlorophyll release in *C. vulgaris* biomass [61]. Similarly, a mechanical disruption treatment was unnecessary when chlorophyll bio-accessibility was considered in *C. vulgaris* biomass, with bio-accessibility values already ranging between 77–84% for untreated biomass [62]. No literature was found on fatty acid release during *C. vulgaris* digestion. 

The lower value observed here for disrupted *N. gaditana* (33.3 ± 6.5%) and disrupted *Scenedesmus* (42.8 ± 5.3%) can be due to an incomplete disruption of the biomass. The ball milling of *Nannochloropsis oculata* previously increased lipid digestibility from 0% (untreated biomass) to 34% (after milling) [10], while high-pressure homogenization (HPH) resulted in a digestibility increase from 36–40% (untreated biomass) to 56–62% (after HPH) in *Nannochloropsis* sp. biomass [9].

Another possible explanation is that a certain degree of lipid hydrolysis by algae lipases, for instance, immediately after harvesting or during the cell disruption treatment, is needed before digestion to facilitate complete lipid hydrolysis by pancreatic lipases. Indeed, it is known that FFA concentrations increase quickly after HPH when *Nannochloropsis* biomass is not immediately cooled [63]. It cannot be inferred what caused the incomplete lipid digestibility for disrupted *N. gaditana* in this study, but it is clear from the literature that cell disruption favors digestibility [9,10]. The same can be expected for the *Scenedesmus* case, since its recalcitrant cell wall is known to limit the availability of other nutrients, such as carbohydrates [27]. It can be concluded that the studied algae biomass differs significantly in terms of fatty acid composition and that differences in lipid digestibility will also contribute to differences in nutritional value.

### 3.4. Protein Composition and Digestibility

The highest protein levels were found in *C. vulgaris* and disrupted *N. gaditana* (45.5 ± 1.3% and 46.2 ± 0.3%, respectively on dm basis) and the lowest in *P. purpureum* (22.2% ± 0.1%). Amino acids essential to humans ranged between 26 and 28% of the total amino acid content (Table 3).

**Figure 4:**
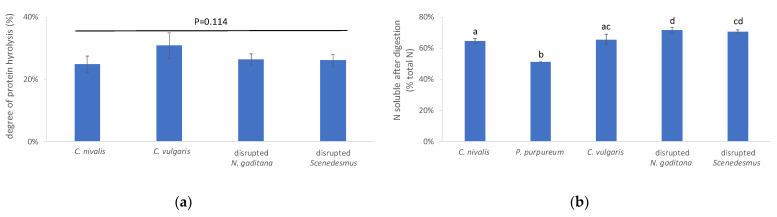
(**a**) N solubility after digestion, and (**b**) degree of protein hydrolysis after digestion. Values shown in the left panel are significantly different when bars are not labeled with a common letter. The type of algae biomass had no significant impact on the degree of protein hydrolysis (*p* = 0.114, 1-way ANOVA). The degree of protein hydrolysis could not be determined for *P. purpureum*.

To examine protein digestibility, three different approaches were followed. First, the concentration of free amino acids after digestion was determined. Yet, the HPAEC profiles of digesta samples contained several interfering peaks, making reliable amino acid quantification impossible. Therefore, a second approach was followed, where the nitrogen fraction that is soluble after digestion was determined (Figure 4a).

The nitrogen solubility after digestion was the lowest for *P. purpureum* (51.1 ± 0.1%) and significantly higher values were noted for *C. nivalis* and *C. vulgaris* (64.5 ± 1.5% and 65.5 ± 3.4%, respectively) and disrupted *Scenedesmus* and disrupted *N. gaditana* (70.4 ± 1.3% and 71.5 ± 1.8%, respectively). The highest absolute soluble nitrogen levels after digestion were observed for *C. vulgaris* and disrupted *N. gaditana* (5.7 mg soluble N/100 g dry algae and 6.0 mg soluble N/100 g dry algae, respectively.

The lower N solubility after the digestion of *P. purpureum* might be related to its high polysaccharide content [64], of which some are known to increase medium viscosity [65], possibly affecting protein solubilization and protein–protease interactions. The observed value for *C. vulgaris* (65.5 ± 3.4%) lies within the range found in the literature for heterotrophically cultivated *C. vulgaris* (43–49%) [56,66], 2 commercial *Chlorella* products (60–63%) [56], 11 commercial *Chlorella* products (mean 51 ± 9%) [67] and a commercial *C. vulgaris* product (76%) [12]. It should be noted that in the last study (Niccolai et al., 2019), an in vitro pig digestion model was used, while the first three studies followed the human digestion consensus protocol also used here. Slightly lower values were reported for untreated *N. oceanica* (50%) by Niccolai et al. [12] and for four untreated *Nannochloropsis* samples (48–59%) by Wild et al. [6], who also used an in vitro pig digestion protocol. Yet, Wild et al. observed a higher value after cell disruption by ball milling (78–80%). Similar values were found in this study for disrupted *N. gaditana* (71.5 ± 1.8%) and disrupted *Scenedesmus* (70.4 ± 1.3%).

Next, the degree of protein hydrolysis was assessed. It should be noted that a 100% conversion to primary amines cannot be expected, since algae contain low levels of non-protein nitrogen, mainly as inorganic nitrogen, nucleic acids, and chlorophylls [68]. For example, in the case of *C. vulgaris*, 10% of all nitrogen was reported to be non-protein nitrogen [69]. When protein hydrolysis was assessed in digested *P. purpureum*, negative values were obtained. This was probably linked to the viscosity-enhancing effect of *P. purpureum* biomass, which may have affected substrate–enzyme interactions and/or hindered the complete removal of suspended particles by centrifugation. The latter biases the absorption measurement-based analysis due to light scattering from colloidal particles. The degree of protein hydrolysis of the four other algae ranged between 24.8 ± 2.6% (*C. nivalis*) and 30.8 ± 4.1% (*C. vulgaris*), with no statistical difference among samples (*p* = 0.11, Figure 4b). Using a similar protocol, Cavonius et al. [10] obtained a protein hydrolysis degree of ±35% for disrupted *Nannochloropsis oculata*, while in the untreated biomass, only 3% of the peptide bonds was hydrolyzed. Although this analysis provides only a first indication, it seems that the proteins of the algae shown in Figure 4b are fairly digestible. About 30–35% of all proteins were insoluble after digestion (Figure 4a) and thus indigestible, while 25–30% of all nitrogen was present as free amino groups after digestion (Figure 4b). It can be speculated that at least part of the remaining soluble fraction is also bio-accessible, since brush-border peptidases are not included in the standard digestion protocol used in this study. Brush-border peptidases are required to complete protein and peptide degradation [19] and play an important role in the activation of trypsinogen [20].

### 3.5. Mineral Content

Biomass mineral concentrations (P, K, Ca, Na, Cl, Fe) are listed in Table 4. For all algae, P, along with K, Na, and Cl, is always one of the main minerals. Na and Cl levels vary largely between different algae types, probably due to the use of different addition levels in the growth medium (cfr. Section 2.1, Algae Biomass) and due to the different harvesting types. Fe concentrations are relatively low (<1.5 g Fe/kg) but can still be nutritionally relevant. Indeed, when defatted *Nannochloropsis oceanica* containing 2.6 g Fe/kg biomass was included in pig feed at a 0.5% dosage, it alleviated the anemic status of weanling pigs [70], suggesting that the algae Fe bio-availability was high. Phytic acid (or inositol hexakisphosphate) can play an important role in this regard, since the bio-accessibility of Fe and other minerals is known to be limited by the presence of phytic acid in many staple foods. In cereals, legumes, nuts, and oilseeds, about 60–90% of total phosphorus is part of phytic acid that can bind to cations such as Ca, Fe, K, Mg, Mn, and Zn, making them insoluble and unavailable for uptake [71]. Not only phytic acid, but also other phosphate forms can form insoluble complexes with minerals such as magnesium ammonium phosphate [72]. In the present study, between 45% (disrupted *Scenedesmus*) and 67% (*C. nivalis*) of all phosphorus was present as soluble orthophosphate (PO_4_^3−^) after in vitro digestion (Table 4). At least this fraction is not made insoluble and can potentially be taken up by the body. Accordingly, Wild et al. did not detect inositol phosphate isomers in 16 commercial algae samples, including *Arthrospira*, *Chlorella*, *Nannochloropsis*, and *Phaeodactylum* samples [6]. For *P. purpureum*, the observed soluble orthophosphate value was slightly lower than that of the control. Again, this is possibly linked to the viscosity enhancing effect of *P. purpureum* biomass, as already mentioned in the discussion of the protein hydrolysis results (Section 3.4).

### 3.6. Bioactivity Screening-ACE-1 Inhibition

**Figure 5:**
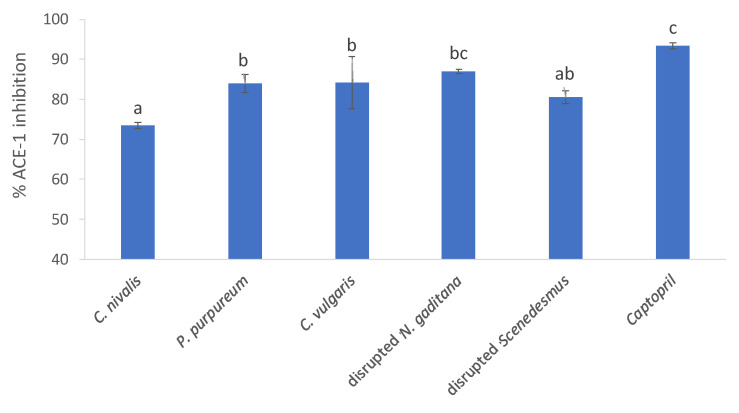
ACE-1 (angiotensin-1-converting enzyme) inhibition by microalgae assayed at a concentration of 1 mg/mL. Values are significantly different when bars are not labeled with a common lowercase letter.

ACE-1 is known to regulate blood pressure and the salt–water balance within many mammalian species. ACE-1 inhibitors work within the renin angiotensin aldosterone system (RAAS). The kidneys produce renin in response to low blood volume and low sodium and high levels of potassium. The enzyme renin, which is the rate limiting step in the RAAS, acts on angiotensinogen and forms the vasodilator Angiotensin I. The enzyme ACE-1 converts Angiotensin I to Angiotensin II, a vasoconstrictor, and this action causes high blood pressure. The inhibition of ACE-1 prevents the formation of the vasoconstrictor Angiotensin II and subsequent high blood pressure or hypertension. An ACE-I inhibition bioassay was performed on the algal biomass. As shown in Figure 5, all microalgae tested inhibited ACE-1 by between 73.4% and 87.1% when assessed at a concentration of 1 mg/mL compared to the positive control Captopril©, which inhibited ACE-1 by 93.48% when tested at a concentration of 0.05 mg/mL.

Disrupted *N. gaditana* biomass was found to inhibit ACE-1 by 87.1%, compared to the control. *C. nivalis*, which inhibited ACE-1 least significantly (73.4%). The percentage ACE-1 inhibition values observed in this study are greater than values reported previously for *Spirulina sp.* (47.6% ACE-1 inhibition) [13]. ACE-1 is a key enzyme that helps to regulate the salt–water balance and blood pressure. The inhibition of ACE-1 is a bioactivity that could be exploited in the development of functional foods for the maintenance of normotensive blood pressure. Algae proteins are likely to be responsible for ACE-1 inhibition, since high ACE-1 inhibition activity was also observed with protein hydrolysates of amongst others *C. vulgaris* and *N. oculata*, as reviewed by Bleakley and Hayes [64].

Although the ACE-1 inhibition assay is an in vitro method, the inhibition of ACE-1 was previously found to directly relate to an observed antihypertensive effect in animal studies. For example, Fitzgerald et al. [73] previously identified an ACE-1 inhibitory tridecapeptide from the macro-alga *Palmaria palmata*. This peptide and the whole alga produced an antihypertensive effect in spontaneously hypertensive rats, when assayed at the same concentrations [74]. The promising data of the current study now justify conducting the necessary experiments to prove anti-hypertensive effects in vivo.

## 4. Conclusions

This study aimed to make an overview of the nutritional characteristics of a set of micro-algae that can be cultivated in the same climatic area. Large differences in organic matter solubility after digestion suggested that for *N. gaditana* and *Scenedesmus* biomass, a prior cell disruption step is needed to avoid the majority of nutrients not being digested. To our knowledge, this is the first study indicating that significant amounts of free glucose can be present after the digestion of *C. nivalis*, *P. purpureum*, and disrupted *Scenedesmus*, which will contribute to the energy content of the biomass. Moreover, it confirms that micro-algae are rich in both essential fatty acids and amino acids, though both concentrations and the degree of digestibility differ among species. The inhibition of ACE-1 was observed for all algae at physiologically relevant concentrations when assayed in vitro against the known ACE-1 inhibitor Captopril^®^. However, future work should include the identification of the active compound responsible for these observed bioactivities (fatty acids, peptides) and confirmation of the potential antihypertensive effect in vivo using an animal model, such as spontaneously hypertensive rats. The results of this study can support algae cultivators and processors in selecting the most appropriate algae species for a particular food or feed application.

## Figures and Tables

**Table 1:** Biochemical composition.

	*C. nivalis*	*P. purpureum*	*C. vulgaris*	Disrupted *N. gaditana*	Disrupted *Scenedesmus*
Carbohydrates (% dm) ^1^	38.4 ± 0.5	51.2 ± 1.9	16.0 ± 3.2	15.8 ± 0.4	45.4 ± 6.9
Proteins (% dm) ^1^	33.4 ± 0.9	22.2 ± 0.1	45.5 ± 1.3	46.2 ± 0.3	25.7 ± 4.4
Lipids (% dm)	22.2 ± 0.4	13.2 ± 0.9	21.8 ± 0.3	31.9 ± 0.6	17.5 ± 0.5
Ash (% dm)	3.6 ± 0.1	8.4 ± 0.1	8.2 ± 0.3	6.4 ± 0.0	3.0 ± 0.1

^1^ Carbohydrates were calculated as the sum of all monosaccharides and uronic acids, while proteins were calculated as the sum of all amino acids, each corrected for water uptake during hydrolysis.

**Table 2:** Fatty acid concentrations (mg/g dry algae) in non-digested algae biomass. LA = linoleic acid, ALA = α-linolenic acid, EPA = eicosapentaenoic acid, DHA = docosahexaenoic acid, SFA = saturated fatty acids, MUFA = mono-unsaturated fatty acids, PUFA = poly-unsaturated fatty acids.

Fatty Acid Levels (mg/g Dry Algae)	*C. nivalis*	*P. purpureum*	*C. vulgaris*	Disrupted *N. gaditana*	Disrupted *Scenedesmus*
C12:0	0.15 ± 0.01	0	0.25 ± 0.03	0.44 ± 0.04	0.20 ± 0.01
C14:0	0.27 ± 0.02	0.04 ± 0.04	0.36 ± 0.01	4.05 ± 0.08	0.20 ± 0.01
C16:0	17.03 ± 0.74	14.83 ± 2.18	23.89 ± 0.95	17.87 ± 0.73	14.06 ± 0.41
C16:1 (cis 9)	0.41 ± 0.35	0.04 ± 0.04	0	31.49 ± 0.97	0.24 ± 0.01
C17:0	4.75 ± 0.28	0	2.21 ± 0.11	0.23 ± 0.01	1.24 ± 0.04
C18:0	0.96 ± 0.07	0.70 ± 0.12	1.70 ± 0.07	0.34 ± 0.02	0.94 ± 0.03
C18:1 (cis-9)	20.88 ± 1.08	1.82 ± 0.50	28.26 ± 1.08	1.94 ± 0.12	16.68 ± 0.77
C18:1 (cis-11)	11.15 ± 0.59	0.16 ± 0.03	1.84 ± 0.06	0.27 ± 0.01	1.11 ± 0.03
C18:2 (cis,cis-9,12) or LA	12.57 ± 0.83	4.62 ± 0.18	15.21 ± 0.78	1.37 ± 0.08	8.54 ± 0.49
C18:3 (cis 6,9,12)	0	0.14 ± 0.08	0.70 ± 0.06	0.82 ± 0.07	0.31 ± 0.07
C18:3 (cis 9,12,15) or ALA	26.98 ± 2.07	0.57 ± 0.09	22.39 ± 1.42	0.08 ± 0.03	12.06 ± 0.61
C20:0	0	0	0	0.11 ± 0.01	0.12 ± 0.03
C20:1 (cis11)	0	0	0.50 ± 0.15	0	0.33 ± 0.03
C20:2 (cis 11,14)	0	0.29 ± 0.04	0	0	0
C20:3 (cis8,11,14)	0	0.55 ± 0.08	0	0.90 ± 0.09	0
C20:4 (cis 5,8,11,14)	0	8.24 ± 1.52	0	3.16 ± 0.27	0
C22:1 (cis 13)	0	0	0.15 ± 0.04	0.44 ± 0.02	0.09 ± 0.01
C20:5 (cis 5,8,11,14,17) or EPA	0	3.61 ± 0.76	0	54.87 ± 4.67	0
C22:6 (cis 4,7,10,13,16,19) or DHA	0	0	0	0.20 ± 0.03	0
Sum unidentified peaks	23.03 ± 1.69	1.40 ± 0.54	25.50 ± 1.93	20.79 ± 1.41	16.41 ± 0.94
omega-3 fatty acids	27.0 ± 2.1	4.2 ± 0.8	22.4 ± 1.4	55.1 ± 4.7	12.1 ± 0.6
omega-6 fatty acids	12.6 ± 0.8	13.8 ± 1.5	15.9 ± 0.8	6.2 ± 0.3	8.9 ± 0.5
sum SFA	23.2 ± 0.8	15.6 ± 2.2	28.4 ± 1.0	23.0 ± 0.7	16.8 ± 0.4
sum MUFA	32.4 ± 1.3	2.0 ± 0.5	30.8 ± 1.1	34.1 ± 1.0	18.5 ± 0.8
sum PUFA	39.6 ± 2.2	18.0 ± 1.7	38.3 ± 1.6	61.4 ± 4.7	20.9 ± 0.8

**Table 3:** Amino acid composition.

	*C. nivalis*	*P. purpureum*	*C. vulgaris*	Disrupted *N. gaditana*	Disrupted *Scenedesmus*
Sum amino acids (mg/g dm) ^1^	334 ± 9	222 ± 1	455 ± 13	462 ± 3	257 ± 44
Sum essential amino acids (mg/g dm)	100 ± 1	71 ± 4	146 ± 2	150 ± 2	77 ± 14
Arginine (mg/g dm)	94 ± 1	61 ± 3	128 ± 11	138 ± 8	69 ± 4
Lysine (mg/g dm) ^2^	17 ± 0	12 ± 1	23 ± 1	27 ± 1	14 ± 5
Alanine (mg/g dm)	22 ± 1	17 ± 2	38 ± 1	34 ± 1	21 ± 5
Threonine (mg/g dm) ^2^	17 ± 0	10 ± 0	22 ± 1	22 ± 0	15 ± 2
Glycine (mg/g dm)	17 ± 0	12 ± 2	27 ± 1	24 ± 1	15 ± 4
Valine (mg/g dm) ^2^	25 ± 1	15 ± 3	27 ± 0	28 ± 1	14 ± 5
Serine (mg/g dm)	16 ± 0	12 ± 0	18 ± 1	19 ± 0	14 ± 1
Proline (mg/g dm)	16 ± 0	9 ± 0	33 ± 0	34 ± 0	14 ± 3
Isoleucine (mg/g dm) ^2^	13 ± 1	11 ± 0	19 ± 0	22 ± 1	9 ± 4
Leucine (mg/g dm) ^2^	28 ± 0	19 ± 0	41 ± 0	41 ± 1	19 ± 11
Methionine (mg/g dm) ^2^	2 ± 0	5 ± 1	8 ± 0	7 ± 1	3 ± 1
Histidine (mg/g dm) ^2^	6 ± 0	3 ± 0	11 ± 0	10 ± 0	4 ± 1
Phenylalanine (mg/g dm) ^2^	17 ± 0	10 ± 0	24 ± 0	23 ± 0	14 ± 4
Glutamate and glutamine (mg/g dm)	45 ± 6	24 ± 2	52 ± 1	50 ± 0	37 ± 4
Aspartate and asparagine (mg/g dm)	37 ± 2	23 ± 1	40 ± 1	40 ± 1	27 ± 3
Cystine (mg/g dm) ^3^	2 ± 0	0 ± 0	3 ± 0	2 ± 0	1 ± 1
Tyrosine (mg/g dm)	10 ± 0	9 ± 0	16 ± 0	16 ± 0	8 ± 1

^1^ corrected for water uptake; ^2^ amino acids essential in humans; ^3^ cysteine is converted to cystine during HPAEC analysis.

**Table 4:** Mineral concentrations of the initial biomass and soluble orthophosphate concentrations after digestion.

	*C. nivalis*	*P. purpureum*	*C. vulgaris*	Disrupted *N. gaditana*	Disrupted *Scenedesmus*
P (mg/g dm)	7.20 ± 0.11	7.73 ± 0.08	10.44 ± 0.16	13.35 ± 0.15	5.19 ± 0.04
K (mg/g dm)	5.27 ± 0.10	9.84 ± 0.26	7.17 ± 0.03	13.35 ± 0.30	7.59 ± 0.20
Ca (mg/g dm)	3.99 ± 0.04	8.72 ± 0.17	1.04 ± 0.01	3.05 ± 0.10	1.81 ± 0.05
Na (mg/g dm)	1.07 ± 0.03	2.91 ± 0.09	17.11 ± 0.21	3.39 ± 0.10	0.13 ± 0.00
Cl (mg/g dm)	0.94 ± 0.03	1.75 ± 0.10	18.57 ± 0.35	3.03 ± 0.01	0.47 ± 0.02
Fe (mg/g dm)	0.10 ± 0.00	0.11 ± 0.01	1.12 ± 0.00	0.29 ± 0.00	0.37 ± 0.00
After digestion					
Soluble orthophosphate (mg P/g dm)	4.83 ± 0.28	- ^1^	6.03 ± 1.94	7.25 ± 0.92	2.35 ± 0.08

^1^ Soluble orthophosphate concentration after *P. purpureum* digestion was lower than that of the control.

## Supplementary Materials

The following are available online at https://www.mdpi.com/article/10.3390/foods10071516/s1, Table S1: monosaccharide and uronic acid concentrations in initial biomass (no digestion).
