# Supplementary material for: Nutritional Profiling and Preliminary Bioactivity Screening of Five Micro-Algae Strains Cultivated in Northwest Europe
Source: Foods. 2021 Jul 1;10(7):1516. doi: 10.3390/foods10071516 (PMC8307025; doi:10.3390/foods10071516)
Supplement: Supplementary file 1 [file foods-10-01516-s001.zip › foods-1254314-supplementary.pdf]

foods

MDPI

Table S1. Monosaccharide and uronic acid concentrations in initial biomass (no digestion).

	C. nivalis	*P. purpureum*	*C. vulgaris*	Disrupted *N. gaditana*	Disrupted *Scenedesmus*
Sum monosaccharides[1] (mg/g dm)	384.3 ± 5.3	512.2 ± 19.2	160.0 ± 32.0	157.9 ± 3.5	454.4 ± 69.4
Mannitol (mg/g dm)	0.5 ± 0.2	0.3 ± 0.1	0.7 ± 0.7	9.1 ± 0.4	0.5 ± 0.2
Fucose (mg/g dm)	2.4 ± 0.2	0.7 ± 0.1	2.1 ± 0.5	1.9 ± 0.0	5.0 ± 0.0
Rhamnose (mg/g dm)	13.7 ± 0.1	0	16.4 ± 2.8	7.3 ± 0.2	5.0 ± 0.0
Arabinose (mg/g dm)	48.3 ± 0.2	0	15.7 ± 2.9	1.1 ± 0.1	1.1 ± 0.0
Galactose (mg/g dm)	54.6 ± 0.9	100.2 ± 36.4	32.1 ± 7.0	26.9 ± 1.0	32.3 ± 0.5
Glucose (mg/g dm)	280.7 ± 7.3	380.6 ± 13.5	71.4 ± 12.3	93.6 ± 2.3	368.1 ± 76.4
Xylose (mg/g dm)	17.9 ± 0.9	92.0 ± 33.9	10.4 ± 3.2	12.3 ± 1.1	72.3 ± 0.5
Fructose (mg/g dm)	4.2 ± 0.8	0.6 ± 0.0	9.9 ± 2.6	4.7 ± 0.1	11.2 ± 0.2
Ribose (mg/g dm)	3.5 ± 0.4	2.8 ± 0.7	15.8 ± 2.8	16.3 ± 0.1	3.8 ± 0.2
Galacturonic acid (mg/g dm)	0	37.2 ± 13.5	0.2 ± 0.4	0	0
Glucuronic acid (mg/g dm)	2.9 ± 0.0	4.6 ± 0.3	4.3 ± 0.7	3.0 ± 0.1	7.4 ± 0.0
Mannuronic acid (mg/g dm)	0.1 ± 0.0	0.2 ± 0.0	0.7 ± 0.1	0.5 ± 0.0	0.2 ± 0.0

[1] Corrected for water uptake.
